# S228. TOWARD EARLY DETECTION OF TREATMENT RESISTANT SCHIZOPHRENIA: PREDICTIVE INFORMATION ON NON-RESPONSE TO ANTIPSYCHOTICS BY EVALUATION OF A FEW CLINICAL FACTORS: A STUDY BY ROC CURVE ANALYSIS AND CONFIRMATORY MULTIVARIATE ANALYSIS

**DOI:** 10.1093/schbul/sby018.1015

**Published:** 2018-04-01

**Authors:** Felice Iasevoli, Camilla Avagliano, Benedetta Altavilla, Annarita Barone, Mariateresa Ciccarelli, Luigi D’Ambrosio, Danilo Notar Francesco, Eugenio Razzino, Andrea de Bartolomeis

**Affiliations:** University of Naples “Federico II”

## Abstract

**Background:**

Treatment Resistant Schizophrenia (TRS) is associated to poor prognosis and highly disabling course. Early detection of the condition is crucial to rapidly provide targeted interventions. The aim of this study was to evaluate whether it may be possible to distinguish TRS from Antipsychotic Responder Schizophrenia (ARS) patients on the basis of a limited number of measurable clinical factors.

**Methods:**

60 out of 182 eligible patients were included. A multistep diagnostic procedure to separate TRS from ARS was then used. Clinical parameters were recorded. Rating scales were administered, including: the Neurological Evaluation Scale (NES); the Positive and Negative Syndrome Scale (PANSS); the Heinrichs’ Quality of Life Scale (QLS); the UCSD Performance-Based Skills Assessment (UPSA); the Personal and Social Performance (PSP) scale and Specific Level of Functioning (SLOF).

We used the Receiver Operating Characteristic (ROC) curves analysis to distinguish between TRS and ARS. Confirmatory logistic regression and discriminant analysis were additionally used.

**Results:**

Among clinical and demographic parameters, AUCs were significant for previous hospitalizations (AUC=.71; p=.004; SE= .068); antipsychotic dose (AUC=.73; p=.002; SE=.66); duration of illness (AUC=.67; p=.02; SE=.71) and NES score (AUC=.77; p<.0005; SE=.062). Moreover, significant AUCs were found for PANSS Negative subscale score (AUC=.68; p=.013; SE=.068); PANSS total score (AUC=.64; p=.05; SE=.071); QLS score (AUC=.73; p=.003; SE=.067); PSP score (AUC=.69; p=.012; SE=.68); all SLOF areas (AUC ranging from .76 to .68, p<.05), with the exclusion of Area4. A trend toward significance was found for Problem Solving (AUC=.63; p=.08). Among the whole significant variables, the highest specificity for diagnosis was found for NES score and previous hospitalizations (75% and 78.1%, respectively); the highest sensitivity for NES score (71.4%). Accordingly, Odds Ratio of being categorized as TRS were larger for NES score <21.5 (7.5), QLS score <57 (5.49), previous hospitalizations >1.45 and SLOF Area5 <43.5 (4.76 both).

Multivariate analysis supported results of ROC curve analysis. Stepwise logistic regression showed that the following variables were significant predictors of TRS/ARS status: previous hospitalizations, NES score, and antipsychotic dose among clinical variables (χ(3)=27.25, p<.0005, Nagelkerke R2=.48); PANSS Negative subscale score among psychopathology variables (χ(1)=7.75, p=.005, Nagelkerke R2=.16); QLS score among quality of life variables (χ(1)=7.91, p=.005, Nagelkerke R2=.16); SLOF Area2 among social functioning variables (χ(1)=18.05, p<.0005, Nagelkerke R2=.34).

The descriptive discriminant analysis function was significant for clinical variables, χ(6)=23.84, p=.001. The most relevant discriminator variables in this group were NES score, antipsychotic doses, and previous hospitalizations. Discriminant function was also significant for SLOF variables χ(6)=17.67, p=.007, with Area1 and Area3 scores ensuring the highest discriminative power. Discriminant function was only weakly significant for psychopathology and for quality of life variables (PANSS Negative subscale score and QLS score showed the highest discriminative power, respectively).

**Discussion:**

Therefore, the evaluation of a few clinical factors may give solid and predictive information about patient potential to be responsive or non-responsive to antipsychotics. A patient exhibiting a combination of 2 or more lifetime hospitalizations; high NSS; high negative symptoms; low quality of life and psychosocial functioning has low possibility (less than approximately 20%, according to our data) to be responsive to antipsychotic agents.

